# Hospital Meetings

**Published:** 1905-04-08

**Authors:** 


ANNUAL GENERAL MEETING.

### HOSPITAL MEETINGS.

### THE POPLAR HOSPITAL.

The annual general meeting was held at the Poplar Hospital on March 24th. The chair was taken by the Hon. Sydney Holland, who made some interesting and apt remarks on the report for 1904. He drew attention to the fact that King Edward's Fund granted an extra £500 a year on condition that ten more medical beds should be opened. This is important as enabling the nursing staff to have a more complete training. The paying ward, as used before, has been appropriated and thrown open like the others to the sick poor, but a small room for one paying patient has been arranged. This room has been most kindly furnished and decorated at the expense of Mr. Murray, one of the committee.

The hospital finances are in a prosperous condition. As Mr. Holland remarked, annual subscriptions are the life-blood of a hospital, and those to Poplar Hospital have steadily increased. Some saving has been effected in the meat bill, and the expenses for drugs have been less than in former years. The chairman expressed the thanks of the hospital authorities to the matron for her excellent and economical management, and remarked how well the plan of having a lady dispenser had worked. Altogether, with ten more beds, the working expenses of the hospital had only increased £285.

The settled income of the hospital amounts to £3,500. The working men contribute £400 per annum, and it is therefore necessary to collect about £5,000 annually to keep up the yearly amount to £9,000, which are the working expenses for each year. This, Mr. Holland believes, will be obtained in future as it has been in the past. Many votes of thanks were passed, among others one to Colonel Feneran, who has been secretary and house-governor for many years, and who is now retiring.

Proceedings terminated with a vote of thanks to the chairman.

### BRITISH LYING-IN HOSPITAL.

£5,000 Debt on the Nurses' Home.

Mr. C. E. Farmer, presiding at the annual meeting of this hospital on Thursday, 30th ult., drew attention to the fact that the sum of £5,000, expended out of capital for the building of the nurses' home, was still unreplaced, and said that donations would be thankfully received by the board. The home accommodated 22 pupil midwives and nurses, and had been in full use during the year. The deficiency of income caused by the realisation of so large a portion of invested capital was a considerable loss to the hospital, while the additional expense of the home had to be met, and also of the two new wards recently opened. He believed that many ladies who were at present unaware of the existence of the hospital would become subscribers if the good work it was doing among the poor of St. Giles's and the neighbourhood were brought to their notice. During the year 449 in-patients and 618 out-patients were treated, with only one death, and this from phthisis. There was some falling-off in the number of out-patients, owing to extensive demolition of the dwellings of the poor; as, however, the streets were rebuilt, an increase might be confidently looked for, and with this in view two new district midwives had been added to the staff. During the year 28 midwifery pupils and 90 nurses had been trained, and the hospital had been placed by the Central Midwives' Board on the list of approved training schools. He desired to express the warm thanks of the Board to the ladies' committee for their work in visiting the hospital and nurses' home, and in the distribution of the Samaritan Fund, by which very necessitous women were sent to Ramsgate on leaving the hospital.

The report and accounts having been adopted, Colonel W. Aitchison and Mr. A. J. T. F. Aitchison were unanimously elected hon. life governors. The usual votes of thanks closed the proceedings.

### HAMPSTEAD GENERAL HOSPITAL.

The 23rd annual general meeting of the Governors of Hampstead General Hospital was held at the Hampstead Town Hall on March 25th, the Mayor of Hampstead being in the chair.

The annual report and statement of accounts was taken as read.

The Chairman remarked that it was the last time that the general meeting would be held whilst the hospital remained on its old ground, as it was hoped that the new building would be opened during the latter end of May, when a bazaar will be held under the management of the Ladies' Association to assist the building fund. The Out-patients' Department had already been moved to Pond Street, and the Nurses' Home there was already in working order.

He pointed out, further, that an annual sum of £4,500 was required to carry on the charity, and he hoped that the residents in Hampstead would liberally subscribe.

The honorary treasurer, Mr. W. F. Malcolm, proposed a vote of thanks to the Ladies' Association, and asked them to beg still harder this year, as expenses would be considerably increased. As there would be nearly double the number of beds, more money would be necessary.

The re-election of Mr. Malcolm, who has been so generous a benefactor to the hospital, was proposed by Dr. Heath Strange and unanimously passed.

Proceedings terminated with a vote of thanks to Dr. Heath Strange, to whose efforts the bringing about of the new building are largely due, and to the Mayor of Hampstead for presiding.

### SKIN HOSPITAL, STAMFORD STREET.

The annual meeting was held at 52 Stamford Street, S.E., on Tuesday, April 4th, Mr. Lewis B. Sebastian presiding, when the report and accounts were adopted, the honorary officers re-elected, and the usual votes of thanks passed. The report stated that the annual subscriptions had increased, but that a larger reliable income was required to enable the hospital to be rebuilt with the most modern additions. There was an increase of 799 in the number of patients attending during 1904.

### THE INFANTS' HOSPITAL, HAMPSTEAD.

A meeting of the "Infants' Health Society" in connection with the above hospital was held last Wednesday, at which a satisfactory account was given of the work of the hospital during the past year. It was opened nearly two years ago for the treatment of infants under 12 months, suffering from diseases resulting from malnutrition.

The principal feature of the treatment in the hospital is the feeding. Each child has two charts, one showing the exact composition of the food—the relative proportion of fat albuminoids, lactose, etc.—with the number of feeds and amount of each; the other giving details of the child's condition and improvement. The composition of the milk is prescribed by the physician, and the modified milk is prepared at the Walker Gordon Farm. The milk, which is refrigerated immediately on being drawn from the cow to a temperature of 40° F., is afterwards pasteurised.

The modified milk prepared at the farm is supplied in sealed bottles, and the prescribed quantity for 24 hours delivered daily, the empty bottles being collected at the same time. The great objection to this method of infant feeding is the expense—it being impossible to feed a child on the modified milk at a cost under 8d. per day—a price prohibitive to the poorer classes. (One of the aims of the "Infants' Health Society" is to establish milk depôts for the supply of pure and modified milk for infants in various districts.)

The hospital, with the aid of the Infants' Health Society, represents a practical effort towards the spreading of knowledge of the chief factors prejudicially affecting the life and health of infants.

### ROYAL SEA BATHING HOSPITAL, MARGATE.

Earl Derby, presiding at the annual meeting of this hospital on Wednesday, 22nd ult., referred to the increase of £235 in the annual subscriptions, and said that if these were still further augmented a reduction in patients' payments could be made. The total income available for hospital purposes in 1904 fell short of the expenditure by £1,294, and a sale of £1,500 stock was unavoidable. Further accommodation for patients was urgently needed, as there was sometimes a waiting list of 100; and also for the staff, the rooms occupied by the sisters being most unsuitable. During a recent outbreak of scarlet fever the want of proper isolation wards was severely felt. An $x$-ray apparatus had been provided through the kindness of Lady Decies, who raised a fund for the purpose, and excellent results had been obtained. Although, thanks to the medical examiners in London, there had been a reduction in the obviously hopeless cases, there still appeared to be a regrettable tendency to seek admission for cases which had drifted into an incurable condition in other hospitals. By sending these to Margate in the first instance space would be increased in the general hospitals, and the patients would have a more reasonable chance of cure.

With regard to the intention of the Committee to again appeal to the Council of King Edward's Hospital Fund for a grant, he suggested that, while still hoping for success in that direction, efforts to augment the funds from other sources should be redoubled.

Mr. Stewart Sankey, in seconding the adoption of the report and accounts, hoped that the Council's decision, no doubt technically right, might be reversed, since two-thirds of the patients were sent from London, and the King's Fund received support from sources which formerly benefited the hospital.

Sir Henry F. Lennard, in proposing a vote of thanks to Lord Derby, said he had sent a contribution of £1,000 to the hospital.

A sanatorium for Wrexham District is under consideration. During the course of a discussion at a recent meeting of the Sanitary Committee the Mayor said an inquiry he had made showed that town dwellers derived no permanent benefit from sanatorium treatment. One of the councillors suggested that the first step was to get rid of their insanitary property. In spite of this want of encouragement the scheme is to be considered by a small committee.

